# Supplementary material for: Local and thalamic origins of correlated ongoing and sensory-evoked cortical activities
Source: Nat Commun. 2016 Sep 12;7:12740. doi: 10.1038/ncomms12740 (PMC5027246; doi:10.1038/ncomms12740)
Supplement: Supplementary Information — Supplementary Figures 1-8 [file ncomms12740-s1.pdf]

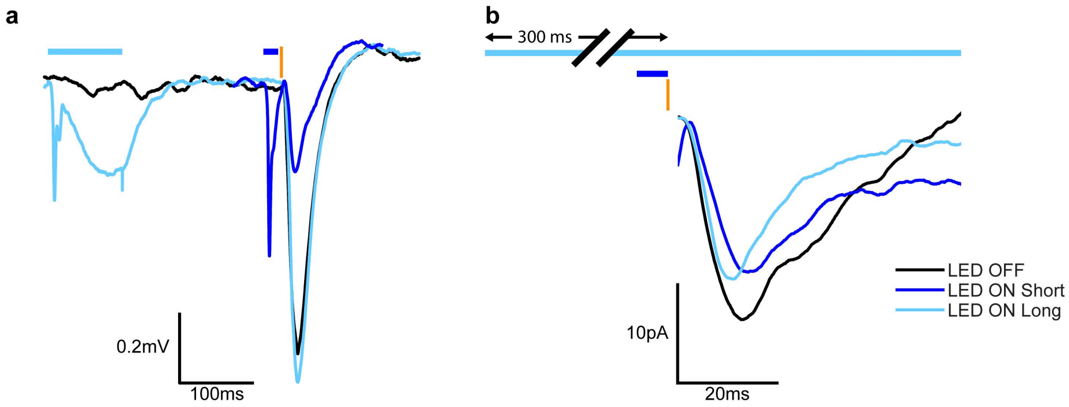

Supplementary Figure 1: Optogenetic isolation of thalamic inputs does not depend on slow effects of illumination. Experiments were designed to find if optogenetic stimulation of GAD cells reduces the EPSC inputs due to slow activation presynaptic GABA(B) receptors in thalamic terminals. (a)**.** Sensory evoked local field potential (LFP) recorded in layer 4 without (black trace) and with (light blue trace) preceding LED illumination (which ended 200ms before whisker deflection). Note that the LED illumination presented 200ms before stimulation had no effect on the response while a short LED illumination presented 5ms before stimulation affected the evoked response (dark blue trace). (b). The duration of LED illumination before whisker stimulation had no effect on the amplitude of the sensory evoked excitatory response. Sensory evoked excitatory response recorded in layer 4 cell in the intact cortex (black trace), 5 ms (blue trace) or 300 ms after LED was switched on (light blue trace). Orange bar marks whisker stimulation.

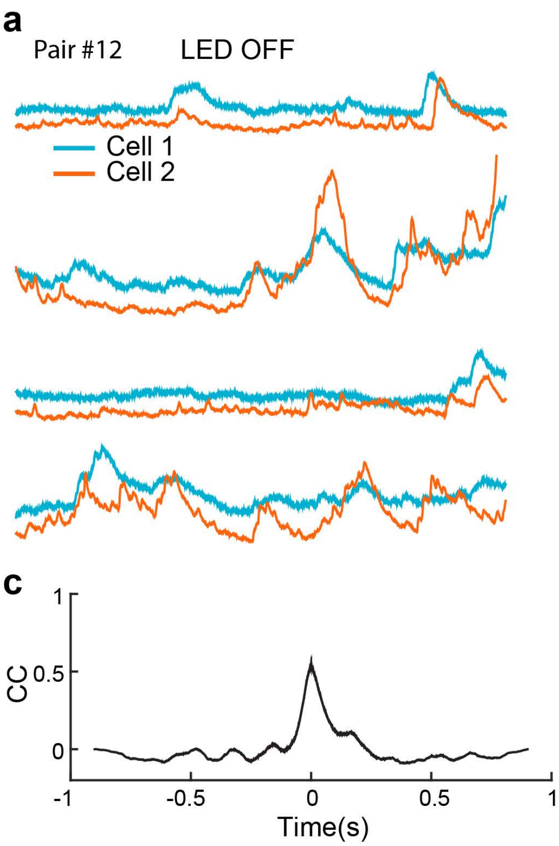

Supplementary Figure 2: Isolation of spontaneous thalamic inputs under current clamp recording mode. (a,b) Example traces of current clamp recordings of L4 cells when the cortex is intact (a) and when cortical firing was silenced (b) in anesthetized mouse. (c,d). The corresponding average cross-correlations between cell 1 and cell 2 for the two conditions.

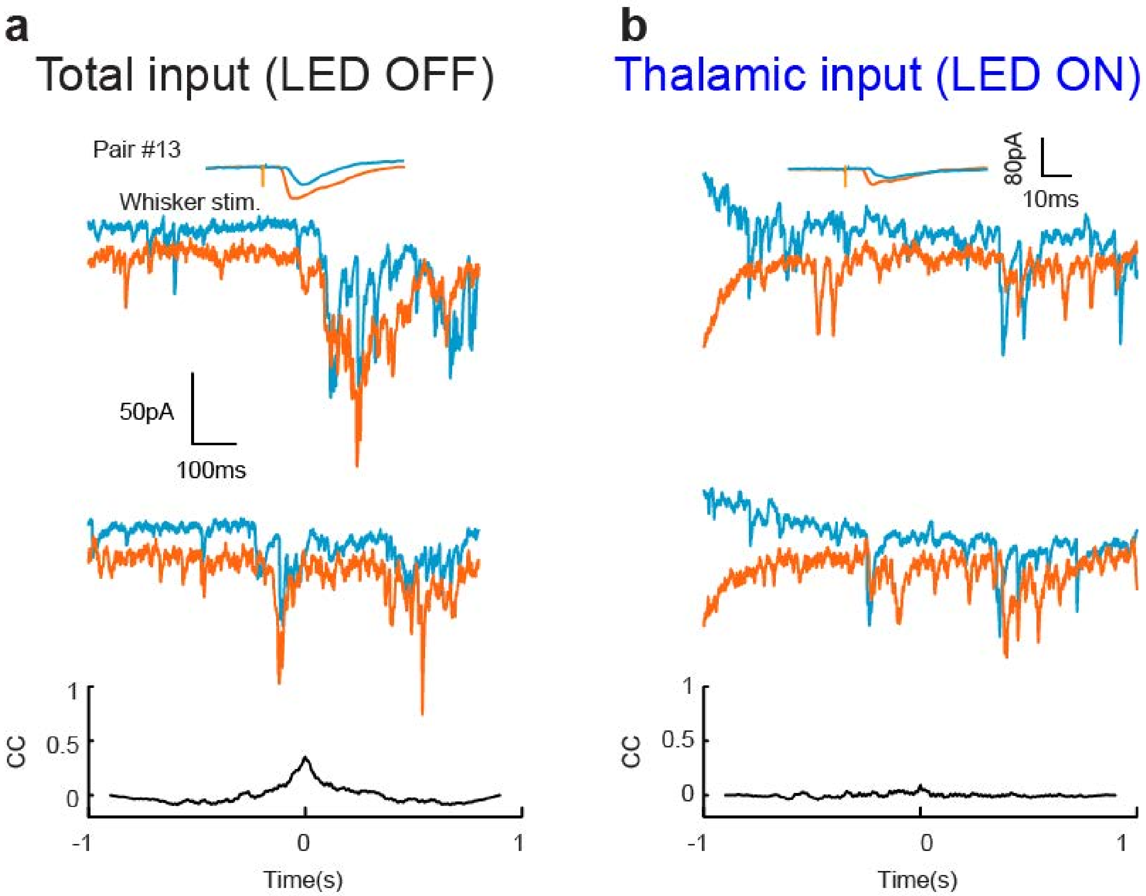

Supplementary Figure 3: Isolation of thalamic inputs of simultaneously recorded two L4 cells during ongoing activity. (a,b) Example traces of voltage clamp recordings when cortex is intact (a) and when cortical firing was silenced (b) in anesthetized mouse. Below are the corresponding average cross-correlations between cell 1 and cell 2 for the two conditions.

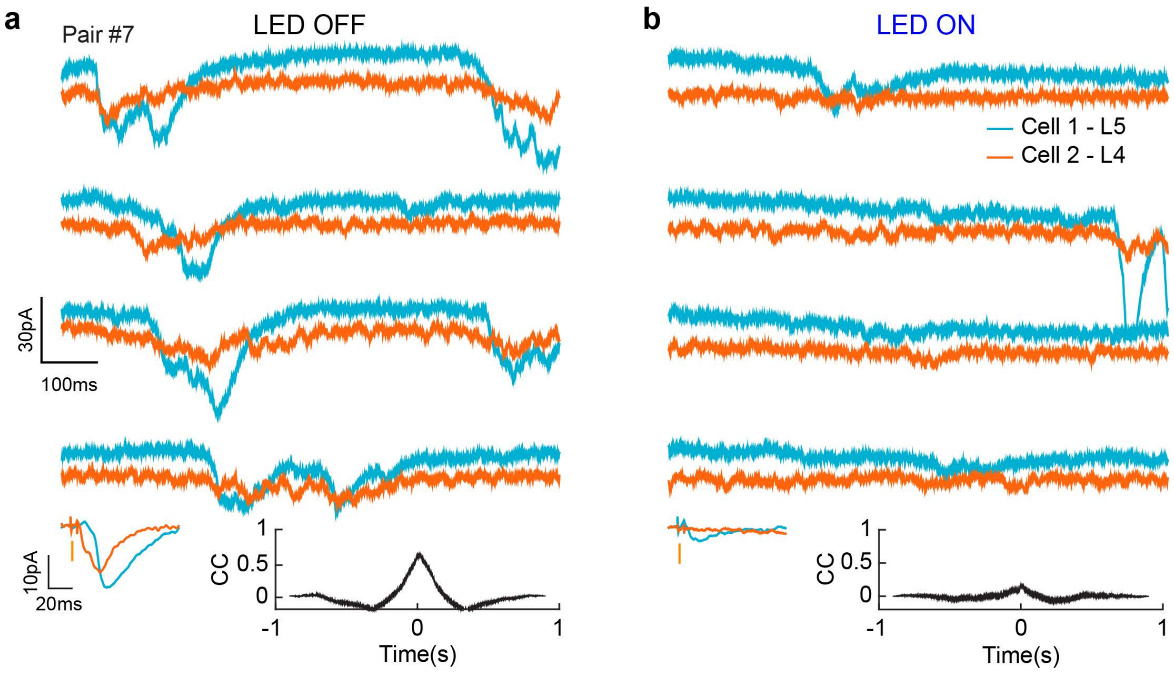

Supplementary Figure 4: Isolation of thalamic inputs of simultaneously recorded L4 and L5 cells during ongoing activity (a,b). Example traces of ongoing activity in two simultaneously recorded L4 (cell 2) and L5 (cell 1) cells when no light was projected (a) and during LED ON condition (b) in anesthetized mouse. Cells were voltage clamped at the measured reversal potential of inhibition. Lower left insets depict the average excitatory currents in response to whisker stimulation of the cells during the same conditions. Lower right insets are the corresponding cross-correlations.

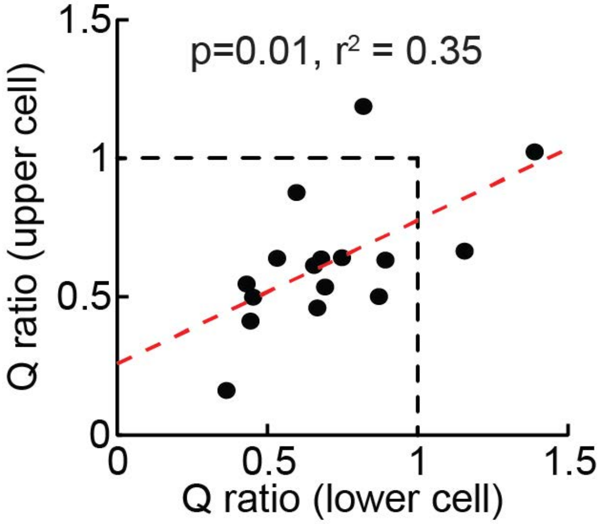

Supplementary Figure 5: Reduction in CC does not result from disappearance of activity in one of the cells. Average change in the charge (Q) during ongoing activity across LED conditions, where the upper cell in each pair is plotted against the deeper cell.

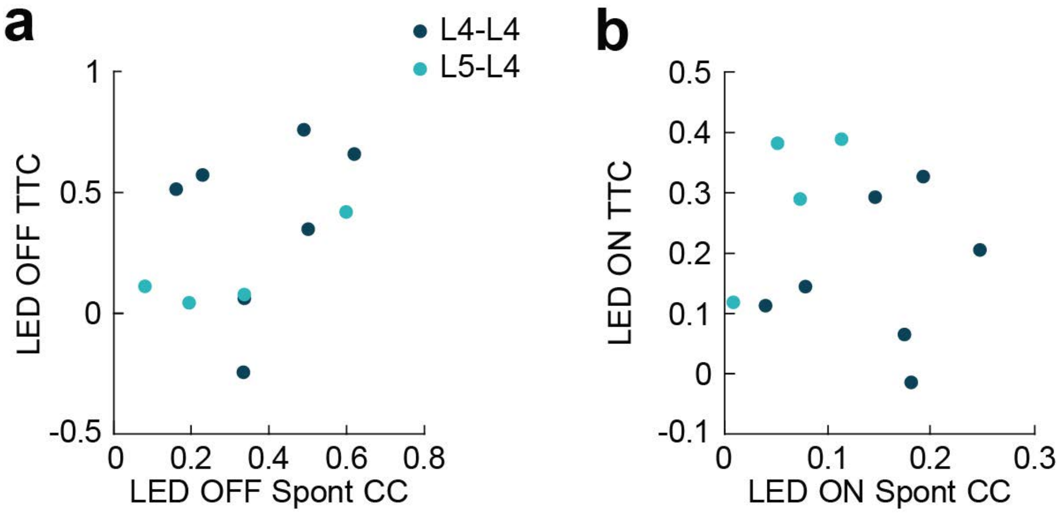

Supplementary Figure 6: TTC_{EE} and CC are not correlated to each other during intact and silenced cortical firing. (a). LED OFF TTC_{EE} plotted against spontaneous LED OFF CC in all recorded pairs (r2= 0.08, p=0.386). (e). Same as (b) but during LED ON condition (r2 = 0.27, p=0.101).

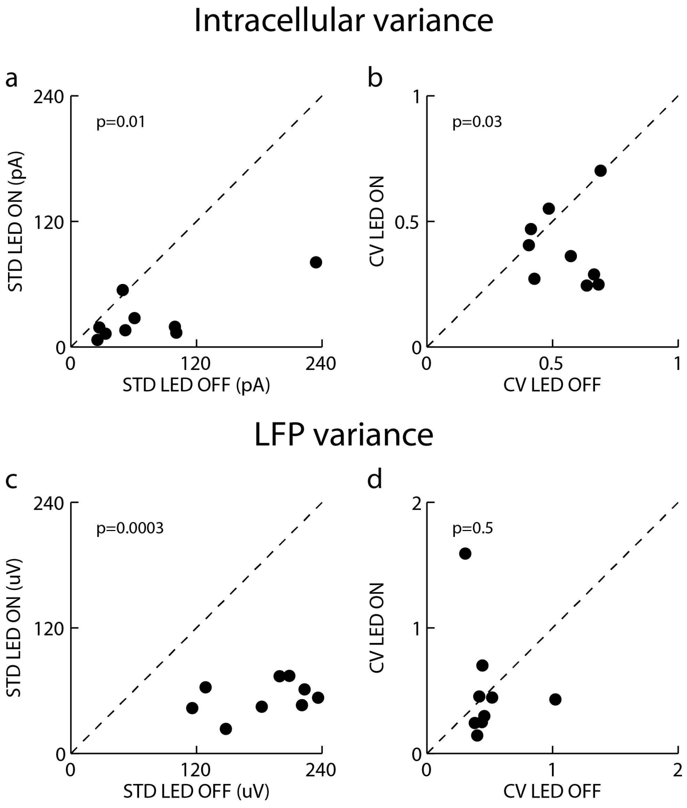

Supplementary Figure 7: The variability of sensory evoked response in awake mice is reduced upon silencing of cortical firing. (a,b). Population data of sensory evoked response standard deviation (a) and coefficient of variation (b) from awake L4 whole-cell voltage clamp recordings (n=9, p = 0.01 and p=0.03, Wilcoxon signed rank test). (c,d) same as (a,b) for LFP measurements in awake animals (n=9, p = 0.0003 and p=0.5, Wilcoxon signed rank test).

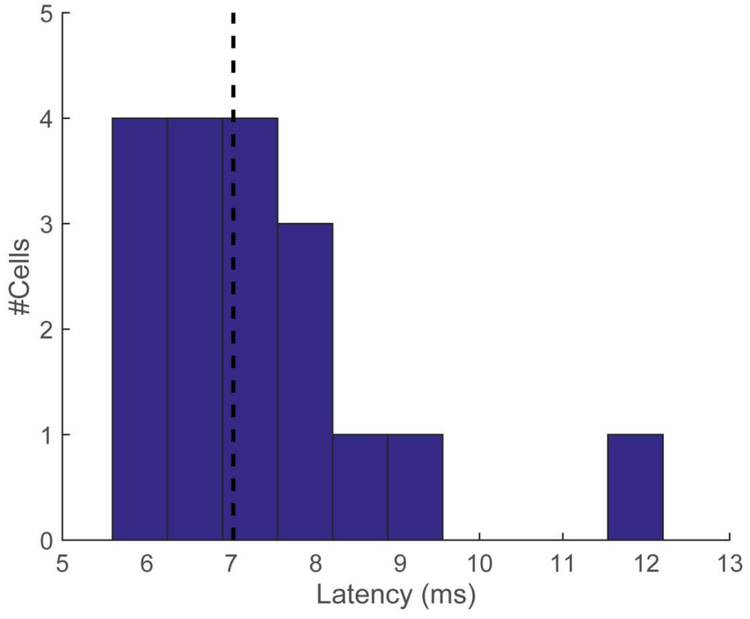

Supplementary Figure 8: Latency distribution of evoked EPSCs of L4 cells. Median is marked by a dashed black line.
